# Supplementary material for: Disentangling metabolic functions of bacteria in the honey bee gut
Source: PLoS Biol. 2017 Dec 12;15(12):e2003467. doi: 10.1371/journal.pbio.2003467 (PMC5726620; doi:10.1371/journal.pbio.2003467)
Supplement: S8 Data — (ZIP) [file pbio.2003467.s008.zip › Z-score_plots/654 microbial product 2,3-Dinor-8-iso prostaglandin F1alpha.pdf]

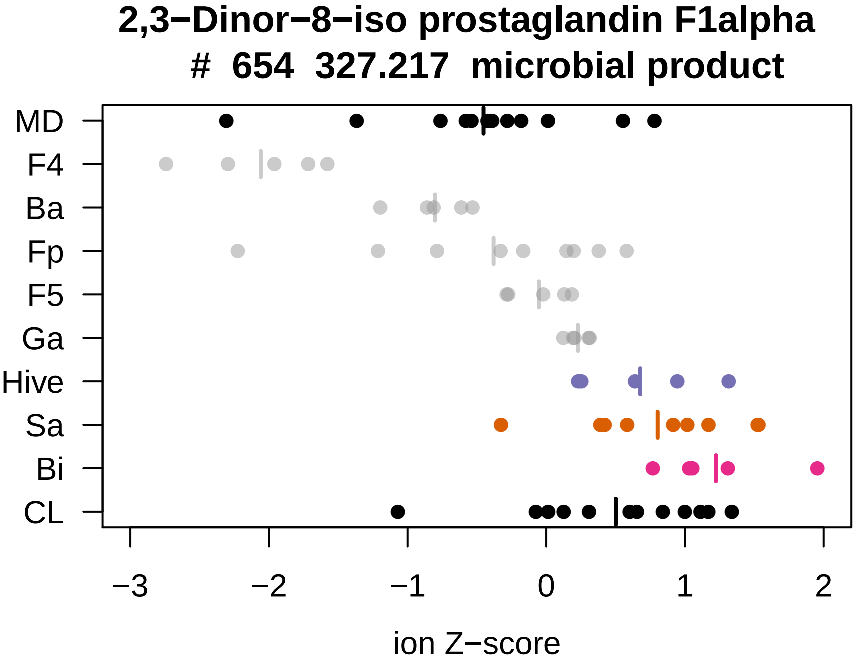

2,3−Dinor−8−iso prostaglandin F1alpha
# 654 327.217 microbial product
MD
F4
Ba
Fp
F5
Ga
Hive
Sa
Bi
CL
−3
−2
−1
0
1
2
ion Z−score
